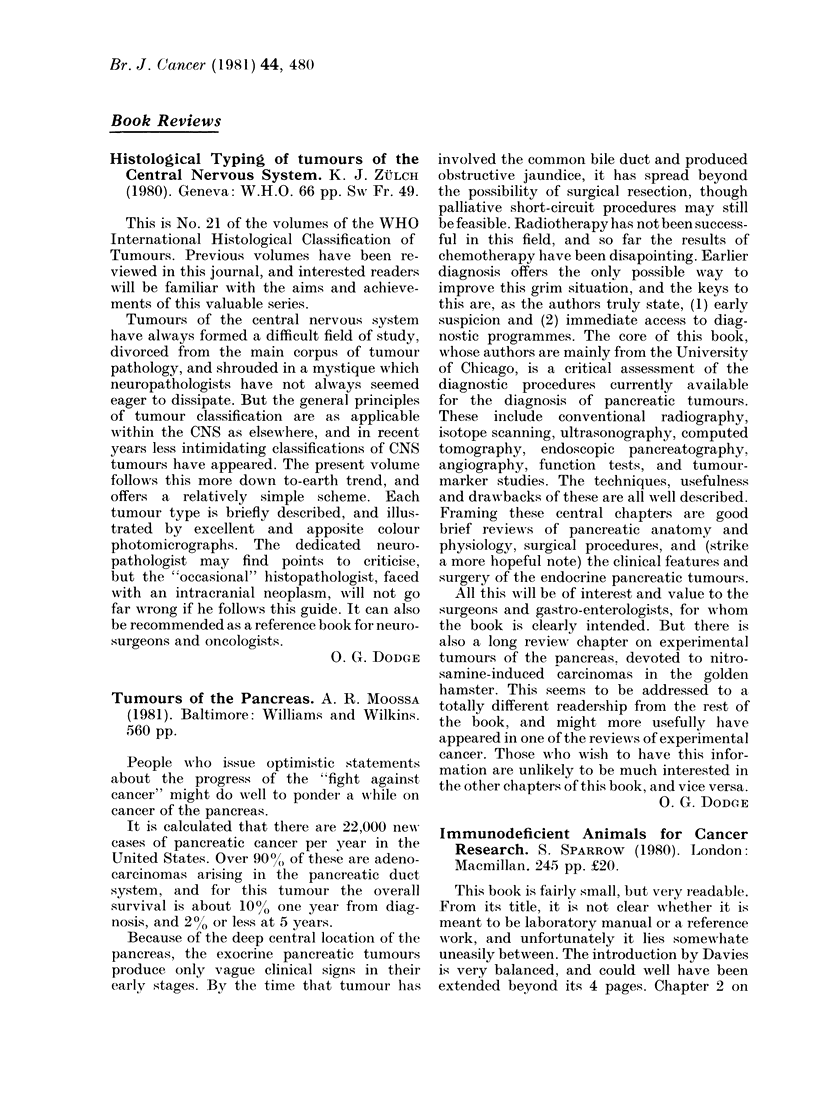# Tumours of the Pancreas

**Published:** 1981-09

**Authors:** O. G. Dodge


O. G. Dodge

**Tumours of the Pancreas.** A. R. Moossa (1981). Baltimore: Williams and Wilkins. 560 pp.

People who issue optimistic statements about the progress of the "fight against cancer" might do well to ponder a while on cancer of the pancreas.

It is calculated that there are 22,000 new cases of pancreatic cancer per year in the United States. Over 90% of these are adenocarcinomas arising in the pancreatic duct system, and for this tumour the overall survival is about 10% one year from diagnosis, and 2% or less at 5 years.

Because of the deep central location of the pancreas, the exocrine pancreatic tumours produce only vague clinical signs in their early stages. By the time that tumour has involved the common bile duct and produced obstructive jaundice, it has spread beyond the possibility of surgical resection, though palliative short-circuit procedures may still be feasible. Radiotherapy has not been successful in this field, and so far the results of chemotherapy have been disapointing. Earlier diagnosis offers the only possible way to improve this grim situation, and the keys to this are, as the authors truly state, (1) early suspicion and (2) immediate access to diagnostic programmes. The core of this book, whose authors are mainly from the University of Chicago, is a critical assessment of the diagnostic procedures currently available for the diagnosis of pancreatic tumours. These include conventional radiography, isotope scanning, ultrasonography, computed tomography, endoscopic pancreatography, angiography, function tests, and tumour-marker studies. The techniques, usefulness and drawbacks of these are all well described. Framing these central chapters are good brief reviews of pancreatic anatomy and physiology, surgical procedures, and (strike a more hopeful note) the clinical features and surgery of the endocrine pancreatic tumours.

All this will be of interest and value to the surgeons and gastro-enterologists, for whom the book is clearly intended. But there is also a long review chapter on experimental tumours of the pancreas, devoted to nitrosamine-induced carcinomas in the golden hamster. This seems to be addressed to a totally different readership from the rest of the book, and might more usefully have appeared in one of the reviews of experimental cancer. Those who wish to have this information are unlikely to be much interested in the other chapters of this book, and vice versa.

O. G. Dodge